# Food-Related Attentional Bias in Individuals with Normal Weight and Overweight: A Study with a Flicker Task

**DOI:** 10.3390/nu12020492

**Published:** 2020-02-14

**Authors:** Francesca Favieri, Giuseppe Forte, Andrea Marotta, Maria Casagrande

**Affiliations:** 1 Dipartimento di Psicologia, Università di Roma “Sapienza”, 00185 Rome, Italy; g.forte@uniroma1.it; 2 Departamento de Psicología Experimental, Universdad de Granada, 18012 Granada, Spain; marotta@ugr.es; 3 Dipartimento di Psicologia Dinamica e Clinica, Università di Roma “Sapienza”, 00185 Rome, Italy

**Keywords:** food-related attentional bias, overweight, normal weight, flicker task, change blindness

## Abstract

The primary purpose of the present study was to investigate attentional biases for food-related stimuli in individuals with overweight and normal weight using a flicker paradigm. Specifically, it was tested whether attention allocation processes differ between individuals with overweight and normal weight using transient changes of food-related and neutral pictures. Change detection latencies in objects of central interest (CI) or objects of marginal interest (MI) were measured as an index of attention allocation in a sample of fifty-three students with overweight/obesity and sixty students with normal weight during a flicker paradigm with neutral, hypercaloric and hypocaloric food pictures. Both groups of participants showed an attentional bias for food-related pictures as compared to neutral pictures. However, the bias was larger in individuals with overweight than in individuals with normal weight when changes were of marginal interest, suggesting a stronger avoidance of the food-related picture. This study showed that food-related stimuli influence attention allocation processes in both participants with overweight and normal weight. In particular, as compared to individuals with normal weight, those with overweight seem to be characterised by a stronger attentional avoidance of (or smaller attention maintenance on) food-related stimuli that could be considered as a voluntary strategy to resist food consumption.

## 1. Introduction

Worldwide, the prevalence of obesity has increased meaningfully over the recent few decades, with nearly 35% of adults classified as overweight and 11% as obese [1]. The emergence of this obesity epidemic has been associated with many factors, including attitudes, habits, cultural bias, beliefs, as well as the environment [2]. The most noticeable environmental change, linked to the higher prevalence of obesity, is the increased availability of food; in particular, the continual exposure to images of food and eating through advertising in magazines and on billboards [3,4]. Attentional biases for unhealthy foods represent one of the most crucial links between food cue exposure and obesity [2].

Attentional bias is a form of a cognitive process involving preferential attention towards relevant stimuli, implicated in the aetiology and maintenance of psychopathology [5]. This particular type of cognitive bias has already been extensively studied in the field of anxiety and mood disorders [6,7] as well as in addiction behaviours [8]. Both in psychological and addiction disorders, attentional bias has been useful to explain why these diseases are self-maintaining and why relapse frequently occurs even after successful treatments.

Equally, studies [9,10,11] have suggested that attentional biases for food cues may play an essential role in the development and the maintenance of maladaptive eating behaviours: food cues become ‘‘attention-grabbing’’ in vulnerable individuals, and they trigger a motivational state of ‘‘wanting’’ that increases the likelihood of behavioural approach and consumption. According to Berridge’s [9] model of food reward, there are individual differences in the sensitivity and responsiveness to the rewarding attributes of environmental food cues [12,13]. In sensible individuals, such as those affected by overweight and obesity [14,15], the exposure to palatable food might produce extreme craving and an impulse to indulge in overeating behaviour, even in the absence of hunger or nutritional deficits [16,17]. According to this theory, empirical research has investigated the existence of food-related attentional bias (FR-AB) in a range of populations with eating-related disorders. These studies have shown that both restrained [16] and external eaters (individuals who eat in response to external food cues) [11,18,19] respond faster to a range of hypercaloric food stimuli relative to neutral (non-food) stimuli. On the other hand, of relevance for the present study, research on attention processes in participants with overweight and obesity is relatively sparse, and it yielded contradictory results [20].

Briefly, participants with overweight and obesity showed more [21,22], equal [23], or less [24] attention for food cues, compared to individuals with normal weight. Individual affected by obesity seem to have a motivational ambivalence towards food, which manifests as an initial orienting of attention towards reward stimuli (e.g., hypercaloric food), and subsequent avoidance of food-related stimuli when attention has to be voluntary maintained [25,26,27,28]. This approach-avoidance pattern has been found by studies using a visual probe task in conjunction with eye-tracking [28] or the P300 wave of evoked potential recording [19].

As underlined by a recent systematic review [29], methodological differences among various studies, such as the type of the task (e.g., Stroop Task; Visual-Probe Task) or the type of the stimuli (pictures or words), may account for differences in observed food-related attentional bias in populations with eating-related disorders (see Table 1). The visual-probe task has been the most widely used task to measure FR-AB. This task involves a pair of stimuli presented simultaneously on different sides of a computer screen followed immediately by a visual probe, which replaces one of the stimuli. Faster response times to the probe that appears in the previous location of a food stimulus compared to a non-food stimulus are suggested to indicate the existence of attentional bias. However, using eye-tracking, some studies demonstrate that this task is limited in its ability to assess attentional bias because some participants showed a tendency to ignore all of the stimuli displayed during the task and to initiate their search only when the probe appeared [19,30].

The second most widely used tool for measuring FR-AB is the modified Stroop task, in which participants are required to identify the colour name of both words indicating food and neutral words [31,32,33]. The Stroop task relies on single word presentations, and it could provide impoverished representations of what might, in real life, give rise to the attentional bias. Therefore, photographs of food may provide a richer, more ecologically valid representation than single words, as they are more indicative of real-life experiences. Furthermore, though the Stroop task is used as a measure of attentional bias, it is not clear whether it represents a measure of stimuli selection or a measure of a response selection [34,35].

More recently, the flicker paradigm for inducing change blindness [36] has been used to measure attentional bias in many pathological diseases, such as addictive behaviours [34,37], or phobic and anxiety disorders [38]. The flicker paradigm is based on the alternation of visual scenes of real life, which differ only for a modified detail (A→A’). This alternation is carried out until the identification of the change by the observer. A blank screen separates the two images, producing a change in luminance that hides the change and prevents the automatic allocation of attention. Change detection latency is assumed to be related to the power of the changed components to capture attention within one single scene.

As the task uses realistic pictures, individuals tend to give priority to some areas of the scene than to others [39]. They usually detect changes in central interest (CI) areas of the scene faster than changes in marginal interest (MI) areas [39]. Both perceptual and semantic characteristics of the visual scene might contribute to creating a sort of priority list that determines what objects are attended to first. Changes in objects of CI involve the gist portion of the pictures, and they are usually detected efficiently [39]. Changes in objects of MI are harder to detect and require a serial visual search. In this case, performance is generally less efficient. Therefore, the flicker task would measure attentional bias for salient target stimuli that capture attention, overcoming limitations of both the Stroop and the visual probe tasks [34]. Moreover, this paradigm could help in the analysis of both automatic and voluntary components of attention, due to the movement of focused attention in the environment (e.g., [38,40,41]). The salience of a visual stimulus influences the exogenous or automatic orienting of the attention, while the subject’s goals drive the endogenous or voluntary orienting of attention (e.g., [42,43]).

Both in psychological disorders and substance abuse/dependence, flicker task has been used to measure attentional bias, and it has helped to explain better why addictive behaviours are self-maintaining. However, to our knowledge, no previous study has used this paradigm to examine the existence of food-related attentional bias in populations affected by overweight or obesity nor has attempted to explain the possible role of this bias in the achievement of maladaptive eating behaviours related to the increase in the body-weight.

The main purpose of the present study was to examine differences in attention for food-related stimuli between individuals with overweight and normal weight through the flicker paradigm. Change detection latencies in objects of central interest (CI) or objects of marginal interest (MI) were measured to either food-related pictures or neutral pictures. We expected that both participants with overweight and normal weight would demonstrate an attentional bias to food-related (hypercaloric food and hypocaloric food) relative to neutral control stimuli, because of the high motivational significance of food. Given the essentiality of food for humans and the oversensitivity of the reward system to food, it is hypothesised that in individuals with overweight, this attentional bias will be significantly enhanced as compared to individuals with normal weight [52,55]. Hypercaloric foods are most attractive because of their highly rewarding and valuable qualities to survival; therefore, we examined whether the attentional bias is restricted to hypercaloric food or whether it is also evident for hypocaloric food.

## 2. Methods

### 2.1. Participants

The participants were 113 Italian undergraduate students (50 men and 63 women; mean age: 24.76 years SD = 2.00), recruited from Sapienza the University of Rome. The inclusion criteria were: (1) absence of eating disorders diagnosis; (2) absence of food allergies; (3) absence of chronic medical diseases; (4) absence of anxiety, depression and other psychopathological disorders; (5) normal or corrected-to-normal vision; (6) absence of colour blindness.

According to body mass index (BMI; Kg/m^2^) [1], participants were divided into two groups: Normal Weight (BMI lower than 25 Kg/m^2^) and Overweight (BMI equal or higher than 25 Kg/m^2^). Fifty-three students were inserted in the group with Overweight (BMI: 29.41; SD = 4.40; Age: 25.02; SD = 2.32); sixty students were included in the group with Normal Weight (BMI: 20.40; SD = 1.22; Age: 24.53; SD = 1.66).

Table 2 shows the main characteristics of the two groups of participants.

### 2.2. Apparatus

An Omron professional digital balance, calibrated in kg, was used to measure the weight. The height of each participant was measured by using a wall-mounted anthropometer. These measures were used to calculate BMI by dividing weight (in kilograms) by height (in meters squared). The WHO [1] indicates the following range of values: underweight (BMI lower than 18.5); normal weight (BMI between 18.5 and 24.9); pre-obesity (BMI between 25.0 and 29.9); obesity class I (BMI between 30.0 and 34.9); obesity class II (BMI between 35.0 and 39.9); obesity class III (BMI equal or higher than 40).

The stimuli of Food Flicker Task were presented using E-Prime 2.0 software on an Intel Core i5 PC, and they were displayed on a 17-inch colour screen. Responses were collected via the computer keyboard.

### 2.3. Visual Stimuli

Twenty-four pictures (640 × 480 pixels) were selected from the International Affective Pictures System (IAPS; [56]: eight neutral scenes, eight hypercaloric foods, and eight hypocaloric foods. Each picture was manipulated using Photoshop**©** software (ver. CS6-13.0) to create an alternative version, removing one detail (49 × 49 pixels) from the scene. According to Rensink [39], changes of Central or Marginal interest were created. A group of other 40 university students (mean age = 22.15, SD = 1.23), who did not participate to the study, viewed each picture for 3 s and generated a written list of scene elements of highest interest. Items chosen by no more than two participants were defined as objects of Marginal Interest (MI); items chosen by all participants were defined as objects of Central Interest (CI). Fifty per cent of the changes referred to MI changing, the other fifty per cent referred to MI changing.

### 2.4. Procedure

**Figure 1:**
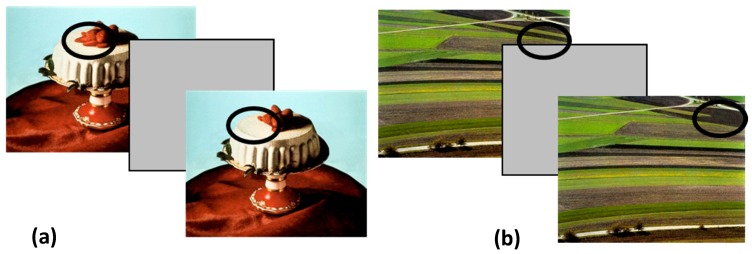
Examples of the stimuli. All the changes were deletion type. In half trials, the changes were of Central Interest (CI; subfigure a) and half of Marginal Interest (MI; subfigure b). The black circle indicates which item appears and disappears during the flicker sequence.

The Local Ethics Committee approved the research (Department of Dynamic and Clinical Psychology—“Sapienza” the University of Rome; prot. 0000197), and it was conducted according to the Helsinki Declaration. Each participant was individually tested in a silent, dimly illuminated room. Before the experimental session, the procedure was thoroughly explained to all participants, and written informed consent was obtained. Subsequently, the participant indicated the current hungry levels on a visual-analogue scale (0–100 mm) and then he/she completed the Food Flicker Task. On each trial of the task, the two versions of the picture repeatedly alternated (240 ms display time), separated by a grey screen (80 ms) (see Figure 1) until the response, consisting in the pressure of the space bar. Then they were required to indicate the change. Three pictures were used for practice, and the twenty-four experimental trials were randomly presented.

After the completion of the task, weight and height were measured.

### 2.5. Data Analysis

Univariate Analyses of Variance (ANOVAs) were carried out to control the differences between groups in age, BMI, and hunger levels. A Group (Normal Weight, Overweight) × Change Type (Central Interest-CI, Marginal Interest—MI) × Stimulus type: (Hypercaloric-food, Hypocaloric-food, or neutral pictures) mixed ANOVA was carried out on both change detection Response Times (RTs) and the number of errors. If the relevant high-order effects were significant, the attentional bias for each type of food-related pictures was calculated as follows:

*Hypercaloric Bias Effect* (RTs detection of changes in Neutral pictures—RTs detection changes in Hypercaloric pictures);

*Hypocaloric Bias Effect* (RTs detection of changes in Neutral pictures—RTs detection of changes in Hypocaloric pictures).

According to the procedure described by Maccari et al. [57], the RTs in the trials in which participants did not detect the change were replaced by the mean RTs + 2.5 SD for that condition. All participants showed a percentage of accuracy greater than 50%.

## 3. Results

### 3.1. Characteristics of the Groups

The two groups did not significantly differ in age (F_1,111_ = 1.67; *p* = 0.20; pƞ^2^ = 0.01) and hungry levels (F_1,111_ = 0.39; *p* = 0.53; Normal Weight: 27.65 vs. Overweight: 30.19).

The ANOVA confirmed the differences between the two groups in the BMI (F_1,111_ = 236.78; *p* < 0.0001; pƞ^2^ = 0.68; Normal Weight: 20.40 kg/m^2^ vs. Overweight: 29.41 kg/m^2^).

### 3.2. Food Flicker Task

#### Detection Response Times

Mean response times and standard deviations are shown in Table 3; attentional bias indices are shown in Table 4.

ANOVA revealed significant main effects for Change Type (F_1,111_ = 333.87; *p* < 0.0001; pƞ^2^ = 0.75) and Stimulus Type (F_1,111_ = 640.58; *p* < 0.0001; pƞ^2^ = 0.85). Overall participants detected MI changes slower than CI changes (20,441 ms vs. 18,761 ms). Moreover, changes in Neutral stimuli were detected slower than changes in the Food-Related stimuli, both Hypercaloric (F_1,111_ = 629.18; *p* < 0.0001; pƞ^2^ = 0.85; 37,618 ms vs. 11,458 ms) and Hypocaloric (F_1,111_ = 744.80; *p* < 0.0001; pƞ^2^ = 0.87; 37,618 ms vs. 9728 ms); however, overall attentional bias for hypocaloric food was greater than attentional bias for hypercaloric food (F_1,111_ = 19.28; *p* < 0.0001; pƞ^2^ = 0.15; 27,890 ms vs. 26,160 ms). No effect of the Group was found (F_1,111_ = 2.95; *p* = 0.08).

The Group × Stimulus Type interaction was significant (F_1,222_ = 4.44; *p* < 0.02; pƞ^2^ = 0.04), showing a higher attentional bias for hypocaloric than hypercaloric food in participants with overweight, although this difference was significant in both the groups (F_1,111_ = 12.34; *p* < 0.001; pƞ^2^ = 0.19, and F_1,111_ = 6.91; *p* = 0.0108; pƞ^2^ = 0.1, respectively).

The Change Type × Stimulus Type (F_1,222_ = 74.37; *p* < 0.0001; pƞ^2^ = 0.4) interaction was also significant, showing a higher attentional bias for hypercaloric food than hypocaloric food when changes were of central interest (F_1,111_ = 87.70; *p* < 0.0001; pƞ^2^ = 0.44; 21,184 vs. 17,888) and an opposite pattern when the changes were of marginal interest, with higher attentional bias for hypocaloric than hypercaloric food (F_1,111_ = 87.70; *p* < 0.0001; pƞ^2^ = 0.44; 21,184 vs. 17,888). Finally, the Group × Change Type × Stimulus Type interaction was significant too (F_1,222_ = 8.31; *p* < 0.001; pƞ^2^ = 0.07). To further understand this interaction, Group × Change type ANOVAs were conducted on each type of attentional bias.

The ANOVA on *hypercaloric attentional bias* showed the main effect of Change Type (F_1,111_ = 27.13; *p* = 0.0001; pƞ^2^ = 0.19), indicating a higher bias when changes of MI than changes of CI occurred. The main effect of Group was only marginally significant (F_1,111_ = 3.88; *p* = 0.051; pƞ^2^ = 0.03). Of relevance, the Group × Change Type interaction was significant (F_1,111_ = 7.58; *p* = 0.007; pƞ^2^ = 0.06). Participants with overweight showed higher hypercaloric attentional bias than participants with normal weight only when changes were of marginal interest (F_1,111_ = 6.47; *p* = 0.012), while no differences were observed when changes of central interest occurred (F < 1).

**Figure 2:**
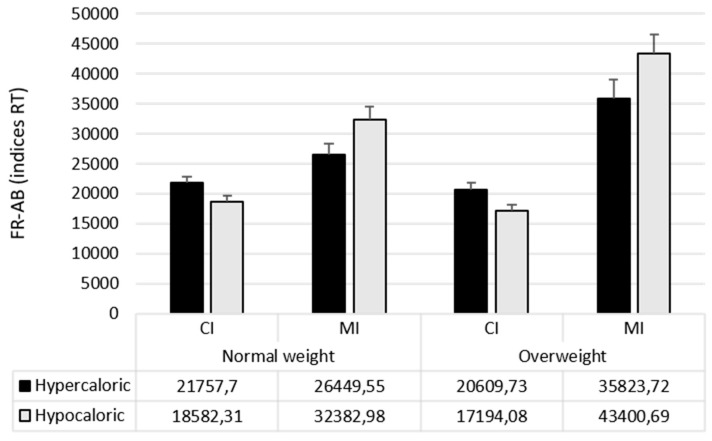
Mean (± SE) of indices of bias considering reaction time in the two groups.

The ANOVA on *hypocaloric attentional bias* showed similar results, with the main effect of Change Type F_1,111_ = 107.63; *p* = 0.0001; pƞ^2^ = 0.49) indicating a higher bias when changes were of MI than when they were of CI. The main effect of Group was also significant (F_1,111_ = 5.55; *p* = 0.02; pƞ^2^ = 0.05), indicating greater hypocaloric attentional bias in individuals with overweight than in normal weight. The Group × Change Type interaction was significant (F_1,111_ = 10.35; *p* = 0.0017; pƞ^2^ = 0.08; see Figure 2). Participants with overweight showed higher hypocaloric attentional bias than participants with normal weight only when changes were of marginal interest (F_1,111_ = 8.84; *p* = 0.0036), while no differences were observed when changes of central interest occurred (F < 1).

### 3.3. Accuracy

Table 2 shows means and standard deviations of the number of errors in the Flicker Task conditions of each group.

The main effects of Group (F_1,111_ = 7.06; *p* < 0.01; pƞ^2^ = 0.06), Change Type (F_1,111_ = 32.61; *p* < 0.0001; pƞ^2^ = 0.23), and Valence of the stimuli (F_2,222_ = 271.12; *p* < 0.0001; pƞ^2^ = 0.71) were significant.

Participants with overweight made more errors than participants with normal weight (0.85 vs. 0.64). In general, the accuracy was worst in MI changes than CI changes (0.91 vs. 0.58). Neutral stimuli allow participants to make more errors than both Hypercaloric (F_1,111_ = 302.80; p < 0.0001; pƞ^2^ = 0.73; 1.58 vs. 0.38) and Hypocaloric (F_1,111_ = 341.04; *p* < 0.0001; pƞ^2^ = 0.75; 1.58 vs. 0.28) stimuli; moreover, more errors in Hypercaloric than Hypocaloric stimuli was found (F_1,111_ = 5.78; *p* = 0.02; pƞ^2^ = 0.05; 0.38 vs. 0.28).

The Change Type × Stimulus Type (F_2,222_ = 6.54; *p* < 0.01; pƞ_2_ = 0.06) interaction was significant, showing a higher number of error for hypercaloric food than hypocaloric food when changes were of marginal interest (F_2,111_ = 12.71; *p* < 0.001; pƞ_2_ = 0.10; 0.66 vs. 0.38).

The Group x Type of Stimulus interaction was also significant (F_1,111_ = 3.36; *p* = 0.04; pƞ^2^ = 0.03), showing a higher number of error in hypercaloric than hypocaloric food stimuli in participants with overweight (F_1,111_ = 5.03; *p* < 0.03; pƞ^2^ = 0.04; 0.46 vs. 0.32), but not in participants with normal weight (F_1,111_ = 1.26; *p* = 0.26).

Finally, the Group × Change Type × Stimulus Type (F_1,111_ = 5.51; *p* < 0.01; pƞ^2^ = 0.05) interaction indicated that attentional bias varied as a function of both the type of the change and the group of participants. To further analyse this interaction, a Group x Change type ANOVA was conducted on each type of attentional bias.

The ANOVA on *hypercaloric attentional bias* showed the main effect of Change Type (F_1,111_ = 8.21; *p* < 0.01; pƞ^2^ = 0.07), indicating a higher bias when changes were of MI than when they were of CI. The main effect of Group did not reach the significance (F_1,111_ = 2.89; *p* = 0.10; pƞ^2^ = 0.02). Of relevance the Group × Change Type interaction was significant (F_1,111_ = 5.60; *p* = 0.02; pƞ^2^ = 0.05). Participants with overweight showed higher hypercaloric attentional bias than the normal weight group only when changes of marginal interest occurred (F_1,111_ = 5.04; *p* = 0.03; pƞ2 = 0.04), while no differences were observed when changes were of central interest (F < 1).

**Figure 3:**
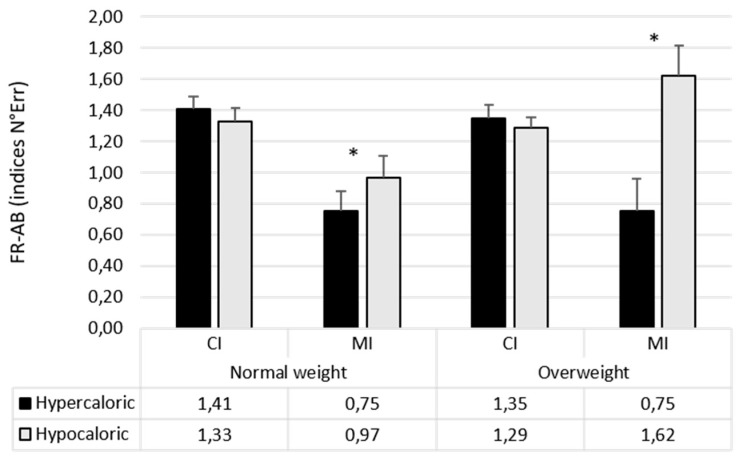
Mean (±SE) of indices of bias considering the number of errors in the two groups of participants (* p < 0.05).

The ANOVA on *hypocaloric attentional bias* showed similar results, with the main effect of Group (F_1,111_ = 4.79; *p* = 0.03; pƞ^2^ = 0.04), indicating higher hypocaloric attentional bias in the group of participants with overweight than in those with normal weight. The main effect of Change Type was not significant (F < 1). The Group × Change Type interaction was significant (F_1,111_ = 9.12; *p* = 0.003; pƞ^2^ = 0.08; see Figure 3). Participants with overweight showed higher hypocaloric attentional bias than participants with normal weight only when changes were of marginal interest (F_1,111_ = 7.98; *p* = 0.01; pƞ2 = 0.07), while no differences were observed when changes of central interest occurred (F < 1).

## 4. Discussion

The results of the present study replicated the main findings regularly observed by using the Flicker task [58,59]. All participants showed a higher change blindness effect and faster detection of CI changes than MI changes. This result is consistent with the assumption that CI changes generate a pop-out effect, leading to an automatic capture of attention. Slower detection in identifying changes in areas of MI can suggest that participants use top-down attentional processing, characterised by a serial visual search strategy [57,60]. If in the area of CI, no change automatically attracts the participant’s attention, a top-down attentional process helps him/her in the detection of changes that the observer expects to identify in the visual scene (i.e., MI areas). This last aspect expresses an active strategy, characterised by serial search, of exploration of the scene to identify new areas where change can occur [61].

Regarding food-related attentional bias, as expected, participants with overweight as well as normal weight showed faster changes detection for food-related pictures than neutral pictures. From an evolutionary perspective, this result is consistent with the view that considers the selective detection of foods as one of the most adaptive characteristics of humans and animals. Moreover, both groups showed a larger attentional bias for hypercaloric food than hypocaloric food when they had to detect changes occurring in the area of central interest. This result may reflect an enhanced automatic orienting towards this type of food. Conversely, the smaller attentional bias for hypercaloric food than for hypocaloric food observed when changes occurred in the area of marginal interest may reflect higher maintenance of attention. This effect could also reflect a delay of attention disengagement from hypercaloric food (the more time attention is maintained on food stimuli, the more time necessary to detect changes of marginal interest). Overall, these findings were observed in both groups and are consistent with the perspective that viewed people as specifically attracted by hypercaloric foods because of their highly rewarding and valuable qualities to survival.

Interesting differences between the two groups of participants were observed when changes occurred in the area of marginal interest. Results showed that participants with overweight presented higher attentional bias for food-related pictures (vs. neutral pictures) as compared to participants with normal weight only when they had to detect changes of marginal interest. This result suggests that individuals with overweight, compared to individuals with normal weight, are inclined to shift attention away from food stimuli faster than from neutral stimuli (less time attention is maintained on food stimuli, less is the time available for detecting changes of marginal interest; consequently, the change detection time increase). This finding is in contrast with the results observed by a previous study that reported increased maintained attention for food cues in individuals with obesity [10]. On the other hand, it is consistent with more recent findings suggesting that some individuals affected by overweight have reduced ability to maintain attention on food cues [27,28].

Overall, the observed pattern of attention allocation in the present study showed a general attentional bias for food-related pictures as compared to neutral pictures in both the groups of participants. This bias was independent by the type of food when participants had to detect CI changes. Conversely, it was smaller for hypercaloric food as compared to hypercaloric food when MI changes occurred. These findings suggest a similar early attentional approach to both types of food and subsequent higher maintenance of attention towards hypercaloric than hypocaloric food. These results could be explained by the higher reward effect of hypercaloric stimuli [9,54], which facilitates automatic visual detection. However, in advanced phases of the attentional process, characterised by a gradual influence of the cognitive control [62], the reward effect of hypercaloric stimuli would make the analysis of the visual scene and the detection of any changes more difficult. The role of cognitive control in the attentional process was underlined by studies, which confirmed the involvement of the frontal lobe, specifically the right dorsolateral prefrontal cortex (DLPF), in the change detection mechanisms [63,64,65]. The role of cognitive control was also proved by the studies revealing the role of dopamine, one of the main neurotransmitters of reward response, in the modulation of frontal cortical activity and its effect in the processes that require focused attention [66]. Frontal areas are involved in executive processes [67], in reward mechanisms [68], in overeating behaviour (for a review see [69]) and also they could be associated in the individual response to food [70,71]. Therefore, an involvement of the same areas in the focused attention could explain the possible modulation affected by stimulus salience on the attentional response.

Finally, the larger attentional bias for food-related pictures as compared to neutral pictures in individuals with overweight than in normal weight when changes of marginal interest occurred, may suggest two different explanations. On the one hand, it could confirm the stronger effect of salience of food stimuli in orienting, focusing attention, and scanning the visual scene [72], which is related to the excessive food intake in individuals with overweight; on the other hand, it could represent an avoidance effect of food-related pictures, reflecting the negative feeling associated to food consumption consequences [73,74]. In conclusion, in the automatic phase of attention might play an important role in the strong desire toward food, whereas when voluntary attentional processing is running, the avoidance of food might represent a strategy to resist food consumption.

### Limits

The literature on the FR-AB is characterised by greater difficulty in giving a clear definition of the construct and obtaining consistent results [24]. This study represents a different point of view in the field of the researches on the FR-AB, and it is the first one that analyses this specific cognitive bias through a Flicker Task. However, some limitations are present.

The small sample size represents a significant limitation because we were not able to detect the differences in FR-AB considering different levels of overweight. It could be useful to highlight whether, with the increase in body weight, there is an increase in the impairment of the attentional process. Also, the small difference in mean BMI between participants with normal weight and overweight could have masked some differences on the FR-AB between the two groups. Moreover, statistical results based on the main effects of the task showed large effects size, confirming the ecological validity of the change blindness assessment. However, these higher effects linked to the small sample size could have influenced statistical power, especially in the interaction effects.

Another limit has been the use of *indices of bias*. This study is the first that used *attentional bias indices* by using the change blindness paradigm, also considering different types of stimuli. For these reasons, we could not compare our results with those of other studies. Further studies are needed to confirm the replicability of the present results that could help in further interpretations of this construct.

## 5. Conclusions

This study showed promising results about the analysis of FR-AB, considering both automatic and voluntary processes of focused attention. Moreover, interesting results emerged also in the analysis of the differences in FR-AB between individuals with normal weight and overweight because they present different pattern of eating behaviours. The Food Flicker Task showed how at different phases of the visual attentional process, the salience of the food stimuli and their characteristics modulate the attentional response. Further studies are needed to detect the relationship between different stages of attentional processes, autonomic response, and the role of prefrontal cortex in response to food stimuli, to try to identify all the processes that could affect our approach to food and food consumption. Knowing all these aspects could help in structuring proper prevention and intervention programs for weight loss, focused on the role of the food stimuli and the adjustment of the individual response to them.

Previous studies (for a review see [75]) showed the potentiality of the Attentional Bias Modification (ABM) in the treatment of maladaptive eating behaviours, but these studies showed higher criticisms [75]. Given longer exposition time to food stimuli compared to the other tasks, Food Flicker Task appears to be a promising ecological approach for analysing focused visual attention and related biases and for structuring interventions that overcome ABM limits (e.g., the short term effect), in order to reduce the maladaptive eating behaviours. Finally, given that individuals with overweight/obesity, compared to those with normal weight, are characterised by higher emotional dysregulation [76], further studies could evaluate whether the emotional regulation of the participants modulates the attentional bias for food-related stimuli.

## Figures and Tables

**Table 1:** Characteristics of the studies considering the attentional bias in individuals with overweight or obesity.

Authors; Year of Publication	Participants	Task	Stimuli Type	Stimuli Duration	Bias	Results
Soetens & Braet [28]	Adolescents with overweight vs. adolescents with normal weight	Imbedded word task	WORDS related to Food or No Food stimuli	N/S *	Attentional Interference	No difference between groups.
Castellanos et al. [10]	Women with normal weight vs. women with obesity (fasting and feeding condition).	Visual Probe Task [Eye tracking]	PICTURES of Food vs. No Food stimuli	2000 ms	Gaze direction bias Gaze duration bias Reaction time bias	Fasting Condition: no differences between groups. Feeding condition: individuals with obesity showed a higher bias than individuals with normal weight.
Calitri et al. [44]	Graduate Students	Food Stroop Task	WORDS related to Healthy Food vs. Unhealthy Food vs. No Food stimuli	Until Participant Response	Cognitive Bias	Cognitive bias predicted the increase in BMI.
Calitri et al. [44]	Graduate Students	Dot Probe Task	WORDS related to Food vs. No Food stimuli	500 ms or 1250 ms	Orienting Attention Sustained Attention	No effects.
Hollitt et al. [45]	Undergraduate student women: Restraint eaters vs. unrestrained eaters	Odd-one-out visual search task	WORDS related to food vs. word relate to neutral stimuli	Until participant response	Speed Detection Disengagement	Higher speed detection of food words in restrained eaters. No differences in the disengagement of attention.
Nijs et al. [27]	Women with overweight/obesity vs. women with normal weight (hunger and satiety conditions)	Visual Probe Task [Eye tracking]	PICTURES of Food vs. No Food stimuli	100 ms or 500 ms	Orienting Attention Maintained Attention Attentional Bias Size	Individuals with normal weight faster than individuals with overweight. Orienting: higher in hunger condition. No differences in Maintained attention or attentional bias between groups.
Phelan et al. [46]	Women with normal weight vs. women with obesity vs. women maintaining weight-loss	Food Stroop Task	WORDS related to hypercaloric food vs. hypocaloric food vs. no food	N/S	Reaction time and Interference-Ratio	Higher reaction time toward hypercaloric food in individuals maintaining weight loss than in the other groups.
Nummenmaa et al. [23]	Graduate students (higher number of females than males)	Visual Search Task [Eye tracking]	PICTURES of hypercaloric food vs. hypocaloric food vs. No food	Until participant response	Orienting Decision Time (after fixation until response)	No association BMI-AB.
Yokum et al. [47]	Adolescent girls (BMI range: 17.3–28.8) 1-year-followup	Food Attentional Network Task	PICTURES of Appetising food vs. Unappetising food vs. No Food	3000 ms	Orienting Reallocation	Faster RTs toward food cue in individuals with higher BMI. Greater AB is associated with a higher increase in weight.
Werthmann et al. [28]	Young women with normal weight vs. young women with overweight/obesity	Visual Probe Task [Eye Tracking]	PICTURES of Highly Palatable Foods vs. No Food	2000 ms	Gaze direction bias; Initial fixation duration bias; Gaze dwell time bias.	Individuals with overweight showed significant Gaze direction bias and shorter Initial fixation duration bias than individuals with normal weight. No differences in Gaze dwell time.
Gearhardt et al. [48]	Women with overweight/obesity	Visual Search Task	PICTURES of Food Low in Fat and/or Sugar vs. Food High in Fat and/or Sugar	N/S	Vigilance Dwell-Time	BMI not related to Dwell-Time. BMI predictor of decreased vigilance.
Loeber et al. [49]	Adults with obesity vs. healthy control	Dot Probe Task	PICTURES of Food vs. No Food	50 ms	Attentional allocation toward stimuli	No differences between groups.
Nathan et al. [50]	Adults with overweight/obesity, assuming placebo vs. adults with overweight/obesity, assuming D2 antagonists	Visual Probe Task	PICTURES of Food vs. No Food	500 ms or 2000 ms	Attentional Bias Toward Food (RTs Probe in no-food-RTs Probe in food)	No differences between groups.
Kemps et al. [51]	Women with obesity vs. women with normal weight	Dot Probe Task	WORDS related to hypercaloric Food vs. hypocaloric Food vs. No Food	500 ms	Attentional Bias	Women with obesity showed higher attentional bias toward food stimuli (faster Reaction time) than women with normal weight Women with obesity showed higher Attentional Bias toward hypercaloric food.
Kemps et al. [51]	Women with obesity (BMI > 30)	Dot Probe Task	PICTURES of Hypercaloric Food vs. hypocaloric Food vs. No Food	500 ms	Attentional Bias	Attentional bias for food cue (faster reaction time).
Schmidt et al. [52]	Adult women with obesity, with or without BED	Spatial Cueing Task	PICTURES of Food vs. No Food	100 ms	Stimulus engagement Stimulus disengagement	Women with BED showed higher engagement than women without BED. No differences in disengagement between groups.
Shank et al. [53]	Children and Adolescents (M/F) with loss of Control of Eating (higher number of participants with obesity) vs. Children and Adolescents with No Loss of Control of Eating (higher number of individuals with normal weight)	Visual Probe Task	PICTURES of High palatable food vs. Low palatable food vs. No Food	2000 ms	Attentional Bias for sustained attention	No relationship between loss of controls eating AB. No relationship between BMI and AB. Loss of Control Eating x BMI: positive relation with AB toward palatable food.
Schmidt et al. [54]	Adolescents with obesity, with and without BED (in both groups the number of females was higher than the number of males)	Visual Search Task	PICTURES of Food vs. No Food	Until participant response	Food detection bias scores	AB higher in individuals with BED than in those without-BED.
Deluchi et al. [21]	Adult individuals with obesity with and without BED (BMI > 35)	Visual Probe Task	PICTURES of Unhealthy Food vs. No Food	SOA: 100, 500, 2000 ms	Orienting Maintenance Disengaging	Orienting AB in both groups; Disengaging AB in individuals with obesity and BED.

* N/S: not specified.

**Table 2:** Characteristics of the groups with normal weight and overweight.

	Normal Weight	Overweight	F	*p*	pƞ^2^
N (M/F)	60 (25/35)	53 (25/28)			
Age	24.53 (1.66)	25.02 (2.32)	1.67	0.20	0.01
BMI	20.40 (1.22)	29.41 (4.40)	236.78	0.0001	0.68
Hungry Level (0–100 visual-analogue scale)	27.65	30.19	0.39	0.53	

**Table 3:** Mean and standard deviation of Response Times (RTs) and accuracy of Flicker Task of the two groups of participants.

		Normal Weight		Overweight	
		Response Time (ms)	Accuracy (n° Errors)	Response Time (ms)	Accuracy (n° Errors)
Central Interest Changes	Neutral cues	26,317 (7710)	1.49 (0.51)	25,014 (8367)	1.46 (0.47)
	Hypercaloric cues	4560 (1824)	0.08 (0.28)	4404 (2202)	0.11 (0.37)
	Hypocaloric cues	7735 (3922)	0.17 (0.42)	7820 (3785)	0.17 (0.43)
Marginal Interest Changes	Neutral cues	44,263 (17,030)	1.27 (1.12)	54,878 (23,218)	2.09 (1.48)
	Hypercaloric cues	17,813 (7082)	0.52 (0.68)	19,054 (9172)	0.81 (0.88)
	Hypocaloric cues	11,880 (5382)	0.30 (0.59)	11,477 (4923)	0.47 (0.72)

**Table 4:** Mean and Standard Deviation of the Attentional Bias Scores in each group.

		Normal Weight	Overweight
Attentional Bias Index (RT)	Food Bias CI	20,170 (7629)	18,902 (7825)
	Food Bias MI	29,416 (15,376)	39,612 (22,797)
	Hypercaloric Bias CI	21,758 (7891)	20,610 (8683)
	Hypercaloric Bias MI	26,449 (15,101)	35,824 (23,591)
	Hypocaloric Bias CI	18,582 (7779)	17,194 (7397)
	Hypocaloric Bias MI	32,383 (16,481)	43,401 (22,738)
Attentional Bias Index (N° Errors)	Food Bias CI	1.37 (1.59)	1.32 (0.49)
	Food Bias MI	0.86 (0.96)	1.45 (1.38)
	Hypercaloric Bias CI	1.41 (0.60)	1.34 (0.62)
	Hypercaloric Bias MI	0.75 (0.98)	1.28 (1.51)
	Hypocaloric Bias CI	1.33 (0.66)	1.29 (0.48)
	Hypocaloric Bias MI	0.96 (1.07)	1.62 (1.39)
